# Outcomes of the Conversion of the Fontan-Kreutzer Operation to a Total Cavopulmonary Connection for the Failing Univentricular Circulation

**DOI:** 10.5935/abc.20180256

**Published:** 2019-02

**Authors:** Gabriel Carmona Fernandes, Guilherme Viotto Rodrigues da Silva, Luiz Fernando Caneo, Carla Tanamati, Aida Luiza Ribeiro Turquetto, Marcelo Biscegli Jatene

**Affiliations:** Instituto do Coração (InCor) - Hospital das Clínicas da Faculdade de Medicina da Universidade de São Paulo (HCFMUSP), São Paulo, SP - Brazil

**Keywords:** Heart Defects Congenital/surgery, Arrihythmias, Cardiac/surgery, Fontan Procedure, Mortality, Fontan-Kreutzer Prodedure

## Abstract

**Background:**

The Fontan-Kreutzer procedure (FK) was widely performed in the past, but in the long-term generated many complications resulting in univentricular circulation failure. The conversion to total cavopulmonary connection (TCPC) is one of the options for treatment.

**Objective:**

To evaluate the results of conversion from FK to TCPC.

**Methods:**

A retrospective review of medical records for patients who underwent the conversion of FK to TCPC in the period of 1985 to 2016. Significance p < 0,05.

**Results:**

Fontan-type operations were performed in 420 patients during this period: TCPC was performed in 320, lateral tunnel technique in 82, and FK in 18. Ten cases from the FK group were elected to conversion to TCPC. All patients submitted to Fontan Conversion were included in this study. In nine patients the indication was due to uncontrolled arrhythmia and in one, due to protein-losing enteropathy. Death was observed in the first two cases. The average intensive care unit (ICU) length of stay (LOS) was 13 days, and the average hospital LOS was 37 days. A functional class by New York Heart Association (NYHA) improvement was observed in 80% of the patients in NYHA I or II. Fifty-seven percent of conversions due to arrhythmias had improvement of arrhythmias; four cases are cured.

**Conclusions:**

The conversion is a complex procedure and requires an experienced tertiary hospital to be performed. The conversion has improved the NYHA functional class despite an unsatisfactory resolution of the arrhythmia.

## Introduction

The Fontan operation (FO) is an important landmark in the history of congenital heart diseases because it increased the life expectancy of children with single-ventricle hearts.^1,2^ After the development of the superior cavopulmonary connection (Glenn operation), the survival rate in univentricular hearts increased leading to the development of FO. The first description by Fontan and Baudet,^3^ was depicted as a right-heart bypass in patients with tricuspid atresia to improve the basal saturation and consequently improve their quality of life and life expectancy while avoiding the complications of chronic hypoxia. These and other techniques that use atrial as a conduit are called atrium-pulmonary connections. Many other techniques and strategies for Fontan operation have been developed since it´s description.

A few years after the first description, in 1973, this technique was modified by Kreutzer,^4^ where the right atrial appendage was connected directly to the trunk of the pulmonary artery with a shorter surgical time than Fontan's previous description. The Fontan-Kreutzer technique (FK) was widely performed and diffused at the beginning, but complications were observed in the long range, such as enlarged atrium, atrial arrhythmias, stasis intracavitary thrombosis and compression of pulmonary veins.^5-9^ These complications are difficult to treat leading to worsening functional class by New York Heart Association (NYHA) and often evolving to ventricular dysfunction and failure of the univentricular circulation.

The next technique, described by de Leval in 1988,^10^, was the cavopulmonary connection using intra-atrial lateral tunnel. In 1990, Marcelletti et al.^11^ described the total cavopulmonary connection (TCPC) using extra-cardiac tube. In subsequent studies it was observed that the TCPC presented better results than the previous techniques.^2,12-16^

Nowadays the TCPC is the most used, however, many patients in whom the old techniques, such as FK, were performed survived and it was possible to observe long-term complications. A treatment option for these patients was to perform a conversion of the FK to TCPC. The removal of the atrium from the pulmonary circulation would decrease the volumetric overload reducing atrial dimensions and consequently lessening secondary outcomes.^17-26^

## Objective

The aim of this study is to evaluate the results of the conversion of FK to TCPC in patients with signs of univentricular circulation failure.

## Methods

A retrospective review of medical records, in-hospital and outpatient notes, was performed for patients who underwent a Fontan conversion (FC). The inclusive criteria consisted of the conversion of FK to TCPC in the period of 1985 to 2016 regardless of their underlying pathology. This was a single center study performed in the Heart Institute (INCOR - HCFMUSP), São Paulo, Brazil. We reviewed all surgical records comprising age at procedure, ventricle morphology, indications for conversion, mortality, the presence of arrhythmias, functional class and the presence of comorbidities after correction.

We excluded the patients in whom FC was indicated but the death occurred before the surgical procedure or intraoperatively, or in whom the procedure was not accepted by the patient or their surrogate decision maker.

This study has been approved by the ethics committee of this Institution by the number CAAE 56617216.6.0000.0068. As the study is retrospective in nature, there was no need for the elaboration of a consent term.

### Statiscal analysis

We used the Kolmogorov-Smirnov test to compare and chooose the sample of the study. Descriptive analysis was performed, including clinical and surgical characteristics. Continuous numerical variables were presented as median and interquartile range (IQR; 25th-75th percentile). Categorical variables were presented as frequencies, absolute number and percentages. Variables with normal distribution were presented average and standard deviation. Estimated actuarial survival were determined using the Kaplan-Meier method. Statistical analysis was performed with SPSS 23.0 for Windows (IBM Corp. Released 2015, IBM SPSS Statistics for Windows, Version 22.0, Armonk, NY: IBM Corp).

## Results

The total number and type of FO performed are shown in Table 1. Of the 18 FK cases, 10 were elected for the conversion to the TCPC due to signs of Fontan circulation failure. All 10 patients previous FK were submitted to a FC procedure and all 10 were included in this study.

**Table 1:** Fontan operation performed between years 1995-2016

Fontan Type	Number of patients
Fontan-Kreutzer	18 (4.3%)
Lateral Tunnel	82 (19.5%)
TCPC with extra cardiac tube	320 (76.2%)
Total	420 (100%)

TCPC: total cavo-pulmonary connection.

The FK were conducted in the beginning of our experience, all were performed before the year 2004, most of them before the year 1999. Only 29 surgeries of lateral tunnels were performed after 2004 and after this year the most performed surgery was the TCPC with extra cardiac tube.

A mortality of 11% (7,9% early deaths and 3,1% of late deaths) was observed for the FO procedure performed in this period. Regarding the ventricle morphology, we observed that 318 cases (75,7%) were classified as left ventricle, 57 (13,6%) as right ventricle, 40 (9,5%) had both ventricles and five (1,2%) had undefined ventricle.

Analyzing the population of the converted, we observed that 40% of the patients were male and 60% female. The youngest patient who underwent conversion was 11 years old and the oldest patient was 42 years old, with the mean average of 23.2 years old.

In nine cases (90%) the surgery was indicated for uncontrolled arrhythmia and one case was indicated by protein-losing enteropathy. In three cases, surgical cryoablation was performed in the same operative time. Before conversion three patients were in functional class I, four in functional class II and three in functional class III.

**Figure 1:**
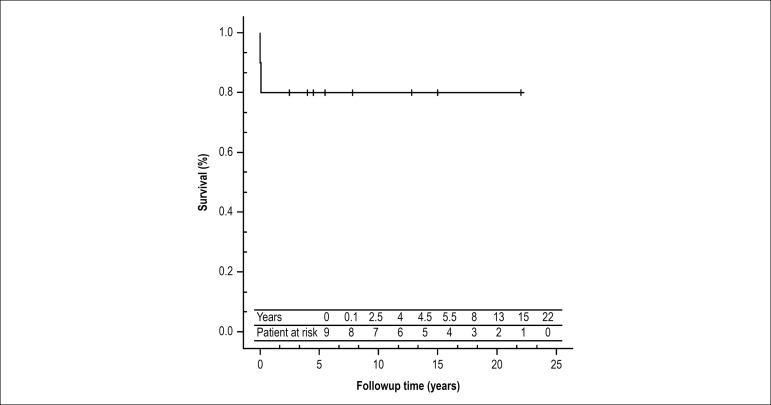
Survival curve of patients submitted to FK conversion to TCPC.

We observed two deaths in the period, an early death (on the second postoperative day) due to significant bleeding and coagulopathy, and a late death (38th postoperative day) due to multiple sepsis and stroke. Both occurred during hospitalization in a postoperative intensive care unit (ICU). The actuarial survival of 5 and 10 years was 80%, as shown in Figure 1.

After conversion, 80% of the patients who were in functional class II or higher evolved with functional class improvement. Currently, six patients are in functional class I (75%), one patient is in functional class II (12.5%) and one patient is in functional class III (12.5%).

Regarding cardiac arrhythmias, 44% of conversions indicated by arrhythmias had improvements after conversion. Four cases were cured with no need of specialist follow-up and three cases had an arrhythmic condition that needed specialist flow-up.

Before conversion, ventricular dysfunction was present in five patients. One of them evolved to death, and all the others had an improvement in their function in relation to the preoperative period, three of which currently have preserved function and one that had had moderate dysfunction previously, and now presents a slight dysfunction. These variables can be visualized on Table 2.

**Table 2:** Clinical improvements after conversion to TCPC

Variables	Before convertion (n = 10)	After convertion (n = 8)
Middly disfuction	2 (20%)	1 (12.5%)
Moderate disfunction	3 (30%)	0 (0%)
Arrhythmias	9 (90%)	4 (44%)
NYHA Functional class I	3 (30%)	6 (75%)
NYHA Functional class II	4 (40%)	1 (12.5%)
NYHA Functional class III	3 (30%)	1 (12.5%)

TCPC: total cavo-pulmonary connection; NYHA: New York Heart Association.

For three of the cases in which surgical cryoablation was performed, one evolved to death despite of the arrhythmia. The other two cases had episodes of arrhythmia after conversion, one of which evolved to bradyarrhythmia requiring a pacemaker, and currently this patient is being evaluated for heart transplantation.

The mean ICU length of stay (LOS) was 13 days, the shortest time was 2 days and the highest 38 days. The average total hospital LOS was 37 days, the shortest being 17 days and the highest 59 days.

As complications, two patients presented bleeding, one pericarditis, one ischemic stroke, one presented convulsive seizures, one presented ventricular dysfunction and one presented bradyarrhythmia. Currently, eight patients are undergoing an outpatient clinic and one patient is being evaluated for heart transplantation.

## Discussion

Fontan-Kreutzer conversion to TCPC is not a simple procedure. Despite a small sample size, we observed a 20% mortality in our experience. The prolonged hospitalization time, average of 37 days, also demonstrates the problems in the management of these patients in the postoperative period. In 25% of the patients evaluated, some types of complications were observed in the postoperative period, where most of them were resolved clinically without the need for new surgical procedures. These facts indicate that ideally this type of surgery should be performed in specialized tertiary centers with the availability of a multidisciplinary team for the best care of the patients.

Caneo et al.^2^ showed a total mortality of 11% for all FO conducted in our Institution, the majority of the death cases were observed in the first period of the study (between years 1984-1994). All atriopulmonary Fontan were performed in the first and second periods (between years 1984-2004), 23,9% of them was elected for conversion years after, and all of these Fontan procedures were performed in the first period. A similar finding was observed in our study, where mortality occurred in the beginning of the experience by the years 1996 and 2000, our first two cases of conversion. It is possible that these two cases have evolved to an unfavorable outcome due to the unavailability of technological resources presented at that time.

Atrial arrhythmias were the main indications of conversion because the modifications performed by Kreutzer resulted in large atrial dilations generating many disorders of the atrial rhythm, which complicated ventricular dysfunction and worsened symptomatology. We obtained an unsatisfactory rate of resolution of these arrhythmias (only 57% of cases indicated by arrhythmia). In cases in which surgical cryoablation was performed (three cases), the outcomes were not favorable: one case evolved to death in the recent postoperative period (due to bleeding and coagulopathy), one arrhythmia was not resolved, and one case progressed with total atrioventricular block, needing definitive pacemaker implantation. This patient evolved with dysfunctions and is currently in line for cardiac transplantation due to significant worsening of functional class and ventricular function. Although most studies suggest a benefit performing cryoablation,^24,26-31^ our findings suggest that surgical cryoablation should not be performed routinely in conversion to TCPC surgery, despite our small sample size.

Studies from South Korea and Japan^32,33^ have reported security and improvement in clinical outcomes by implanting permanent pacemaker in Fontan conversion. However, our only case with pacemaker implantation had unfavorable outcome, and is now in line for heart transplantation. Takeuchi et al.^34^ showed favorable outcomes combining FC with resynchronization, but none of our patients were elected for resynchronization.

The presence of ventricular dysfunction before the FC procedure was found in five cases. All cases were elected to conversion by arrhythmia, one of them died and all the survivors had improved ventricular functions. Therefore, we conclude that the procedure presented a satisfactory result in improving the ventricular function. However, we observed no improvement of the arrhythmia in two cases of the survivors who presented preoperative dysfunction.

There was a significant improvement in functional class and quality of life of these patients after conversion, and therefore, our results demonstrate the importance and necessity of converting selected cases. These findings motivated us to perform this surgery in more cases after our first two cases that evolved to death. Currently, we have only a few cases of FK alive being followed in our ambulatory.

A review by Brida et al.^35^ analyzed 1182 patients from 37 studies and concluded that conversion had substantial mortality risk. However, the results vary between centers and lower early mortality was associated with earlier age and with treatment being performed at high experienced centers.

## Conclusions

The conversion of atrial-pulmonary anastomosis (Fontan-Kreutezer) to TCPC is a complex procedure with high mortality and morbidity justifying a prolonged hospitalization time, so this surgery needs to be performed in experienced tertiary hospitals. The conversion of atrial-pulmonary anastomosis to TCPC has, in our experience, improved the functional class and consequently the patients' quality of life despite an unsatisfactory resolution of the arrhythmia.
